# Association of Prenatal and Perinatal Exposures to Particulate Matter With Changes in Hemoglobin A_1c_ Levels in Children Aged 4 to 6 Years

**DOI:** 10.1001/jamanetworkopen.2019.17643

**Published:** 2019-12-18

**Authors:** Emily C. Moody, Alejandra Cantoral, Marcela Tamayo-Ortiz, Ma. Luisa Pizano-Zárate, Lourdes Schnaas, Itai Kloog, Emily Oken, Brent Coull, Andrea Baccarelli, Martha M. Téllez-Rojo, Robert O. Wright, Allan C. Just

**Affiliations:** 1 Department of Environmental Medicine and Public Health, Icahn School of Medicine at Mount Sinai, New York, New York; 2 Center for Nutrition and Health Research, National Institute of Public Health, Cuernavaca, Mexico; 3 National Council of Science and Technology, Mexico City, Mexico; 4 Division of Community Interventions Research, National Institute of Perinatology, Mexico City, Mexico; 5 Department of Geography & Human Environment, Ben Gurion University of the Negev, Be’er Sheva, Israel; 6 Harvard Medical School and Department of Nutrition, Harvard School of Public Health, Boston, Massachusetts; 7 Department of Biostatistics and Department of Environmental Health, Harvard School of Public Health, Boston, Massachusetts; 8 Department of Environmental Health Sciences, Columbia University Mailman School of Public Health, New York, New York

## Abstract

**Importance:**

Environmental risk factors for childhood type 2 diabetes, an increasing global problem, are understudied. Air pollution exposure has been reported to be a risk factor for this condition.

**Objective:**

To examine the association between prenatal and perinatal exposures to fine particulate matter with a diameter less than 2.5 μm (PM_2.5_) and changes in hemoglobin A_1c_ (HbA_1c_), a measure of glycated hemoglobin and marker of glucose dysregulation, in children aged 4 to 7 years.

**Design, Setting, and Participants:**

The Programming Research in Obesity, Growth, Environment, and Social Stressors (PROGRESS) study, a birth cohort study conducted in Mexico City, Mexico, recruited pregnant women from July 3, 2007, to February 21, 2011, through public health maternity clinics. The present analysis includes 365 mother-child pairs followed up until the child was approximately 7 years of age. This study included data from only study visits at approximately 4 to 5 years (visit 1) and 6 to 7 years (visit 2) post partum because HbA_1c_ levels were not measured in earlier visits. The data were analyzed from March 11, 2018, to May 3, 2019.

**Exposures:**

Daily PM_2.5_ exposure estimates at participants’ home addresses from 4 weeks prior to mothers’ date of last menstrual period (LMP), a marker of the beginning of pregnancy, to 12 weeks after the due date. Exposure was estimated from satellite measurements and calibrated against ground PM_2.5_ measurements, land use, and meteorological variables.

**Main Outcomes and Measures:**

Outcomes included HbA_1c_ levels at 4 to 5 years and 6 to 7 years of age, and the change in the level from the former age group to the latter.

**Results:**

The sample included 365 children, of whom 184 (50.4%) were girls. The mean (range) age of the children was 4.8 (4.0-6.4) years at visit 1, and 6.7 (6.0-9.7) years at visit 2. At the time of delivery, the mean (range) age of the mothers was 27.7 (18.3-44.4) years, with a mean (range) prepregnancy body mass index of 26.4 (18.5-43.5). The mean (SD) prenatal PM_2.5_ exposure (22.4 μg/m^3^ [2.7 μg/m^3^]) was associated with an annual increase in HbA_1c_ levels of 0.25% (95% CI, 0.004%-0.50%) from age 4 to 5 years to 6 to 7 years compared with exposure at 12 μg/m^3^, the national regulatory standard in Mexico. Sex-specific effect estimates were statistically significant for girls (β = 0.21%; 95% CI, 0.10% to 0.32%) but not for boys (β = 0.31%; 95% CI, −0.09% to 0.72%). The statistically significant windows of exposure were from week 28 to 50.6 after the mother’s LMP for the overall cohort and from week 11 to the end of the study period for girls. Lower HbA_1c_ levels were observed at age 4 to 5 years in girls (β = −0.72%; 95% CI, −1.31% to −0.13%, exposure window from week 16 to 37.3) and boys (β = −0.98%; 95% CI, −1.70% to −0.26%, exposure window from the beginning of the study period to week 32.7), but no significant association was found in the overall cohort (β = −0.13%; 95% CI, −1.27% to 1.01%). There was no significant association between PM_2.5_ exposure and HbA_1c_ level at age 6 to 7 years in any group.

**Conclusions and Relevance:**

The findings of this study suggest that prenatal and perinatal exposures to PM_2.5_ are associated with changes in HbA_1c_, which are indicative of glucose dysregulation, in early childhood. Further research is needed because this finding may represent a risk factor for childhood or adolescent diabetes.

## Key Points

**Question:**

Are prenatal and perinatal exposures to fine particulate matter (diameter <2.5 μm) associated with changes in hemoglobin A_1c_ levels in children?

**Findings:**

In this birth cohort study including 365 mother-child pairs, prenatal and perinatal exposures to particulate matter less than 2.5 μm in diameter were associated with lower hemoglobin A_1c_ levels in all children aged 4 to 5 years, and an increase from age 4 to 5 years to 6 to 7 years in girls. A statistically significant gestational exposure window was identified in boys and girls.

**Meaning:**

The findings of this study suggest that prenatal and perinatal air pollution exposure is associated with the risk of altered glucose metabolism during childhood, which could potentially result in an increased risk of diabetes.

## Teaser

This birth cohort study examines the association between prenatal and perinatal exposures to fine particulate matter (diameter <2.5 μm) and changes in hemoglobin A_1c_ levels in children aged 4 to 6 years.

## Introduction

There has been an increase in pediatric type 2 diabetes over the past 2 decades although the occurrence was once rare.^1,2^ This is a public health problem with national and global implications because these trends predict considerable increases in cardiovascular morbidity and mortality for future generations. Disease onset during childhood or adolescence has been shown to have increased long-term health risks compared with onset during adulthood.^3^ Although increasing prevalence of childhood obesity and consumption of energy-dense food are considered to be the most important risk factors for developing childhood type 2 diabetes,^4^ there is growing evidence for the contribution of environmental exposures to the risk of developing this condition^5,6^; however, relatively little is known about such environmental risk factors.

Exposure to components of air pollution, including particulate matter with a diameter less than 2.5 μm (PM_2.5_), has been shown to be associated with increased incidence and progression of type 2 diabetes in adults,^7,8,9^ increased risk of childhood overweight status and obesity,^10,11^ and glucose dysregulation in children.^12^ However, the role of exposure timing in these associations is unclear. There is also increasing evidence for substantial effects of prenatal air pollution exposure on childhood anthropometry, growth, and metabolism.^13^ Recent developments in statistical modeling of air pollution allow for estimations of the time boundaries of critical windows of susceptibility, or developmental stages during which an individual is more susceptible to environmental factors.^14,15,16^ For example, sensitive windows of particulate air pollution exposure have been demonstrated for neurodevelopment,^14^ body composition,^15^ and mitochondrial DNA content in cord blood.^16^ Such methods have yet to be applied to studies of air pollution and childhood diabetes risk. Better understanding of the associations of prenatal PM_2.5_ exposure with metabolic end points leading to diabetes could be gained by examining outcomes by the specific timing of exposure during gestation.

We hypothesized that prenatal and perinatal exposures to PM_2.5_ may be associated with hemoglobin (HbA_1c_) levels or their changes over time in young children. The HbA_1c_ level represents an important investigational target because it serves both as a biomarker of long-term serum glucose levels and a diagnostic criterion for diabetes. Furthermore, we hypothesized that there may be sensitive gestational exposure windows during which PM_2.5_ may be associated with changes in HbA_1c_.

## Methods

### Study Population

In this prospective birth cohort study, the study population consisted of 365 mother-child pairs who were part of the Programming Research in Obesity, Growth, Environment and Social Stressors (PROGRESS) study, which has been described in detail elsewhere.^17^ Pregnant women were recruited in their second trimester between July 3, 2007, and February 21, 2011, through the Mexican Social Security System (Instituto Mexicano del Seguro Social) clinic where they received their primary care. Eligibility criteria were as follows: a gestation period of less than 20 weeks, at least 18 years of age, planning to stay in Mexico City, Mexico, for the next 3 years, access to a telephone, no medical history of heart or kidney disease, no daily alcohol consumption, and no corticosteroid or antiepilepsy medication use. Children and mothers were seen at the research facilities at 1, 6, 12, 18, 24, 48, and 72 months post partum and, as of November 2019, are undergoing follow-up. All mothers provided written informed consent and the institutional review boards at the Harvard School of Public Health, Icahn School of Medicine at Mount Sinai, and the Mexican National Institute of Public Health approved the study, which followed the Strengthening the Reporting of Observational Studies in Epidemiology (STROBE) reporting guidelines.^18^

### Air Pollution Exposure Model

Exposure to PM_2.5_ was estimated using a model developed by Just et al^19^ using moderate resolution imaging spectroradiometer satellite-derived aerosol optical depth measurements. These data were calibrated against ground PM_2.5_ measurements from 12 monitoring stations throughout Mexico City and land use and meteorological variables including roadway density, temperature, relative humidity, planetary boundary height, and precipitation.^19^ Daily PM_2.5_ exposures were estimated across a 1 × 1-km grid over Mexico City, and individual-level exposure estimates were derived using the mother’s home address captured by a handheld global positioning system device. We included daily exposure estimates from 4 weeks prior to the last menstrual period (LMP) to 52 weeks after. Daily exposure estimates were not available for any other copollutants or temperature.

### Hemoglobin A_1c_ Outcome

This study included data from study visits only at approximately 4 to 5 years and 6 to 7 years post partum because HbA_1c_ levels were not measured at earlier visits. These visits will henceforth be referred to as visit 1 and visit 2, respectively. The main outcome variable, the change in HbA_1c_, and the secondary outcomes, HbA_1c_ at visits 1 and 2, were specified prior to beginning the present analysis. A complete case analysis was performed and participants who did not have measured values for HbA_1c_ at both visits 1 and 2 were excluded. The HbA_1c_ was measured in whole blood for all children. Samples were refrigerated and batched to be run twice weekly on an InnovaStar analyzer (DiaSys) at the National Institute of Perinatology, Mexico. The outcome variable for the change in the HbA_1c_ was calculated as a change over time, and recorded as the percentage per year according to the following formula:


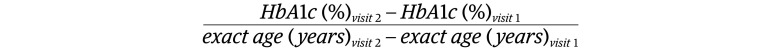
.

### Covariates

The study population was grouped into 3 categories of socioeconomic status (SES) (low, medium, and high) collapsed from the 6-level index created by the Asociación Mexicana de Agencias de Investigación de Mercados y Opinión Pública.^20^ The SES was derived from 13 variables from the prenatal questionnaire. Maternal educational level was assessed by the questionnaire and categorized into 3 groups: did not graduate from high school, high school graduate, and any education after high school. Maternal smoking was not included as a covariate because only 7 mothers in the original cohort (n = 948) reported smoking during pregnancy. Exposure to environmental tobacco smoke was assessed by the questionnaire. Maternal prepregnancy body mass index (BMI), calculated as weight in kilograms divided by height in meters squared, was derived using weight and height measurements taken during the second trimester of pregnancy owing to discrepancies noted in maternal reported prepregnancy weight, as previously described.^21^ Gestational age and birth weight were recorded at the time of delivery but were not included as covariates because they may be on the causal pathway between prenatal PM_2.5_ exposure and HbA_1c_ level in childhood.^22^ Because ultrasonography was not routinely performed as the standard of care, gestational age was based on the LMP and by a standardized physical examination to identify the gestational age at birth.^23^ When the physical examination assessment of the gestational age differed by more than 3 weeks from the gestational age based on LMP, the physical examination was used in lieu of the LMP-calculated gestational age.^21^ The covariates considered in the model included child sex, exact child age at visit 1, maternal prepregnancy BMI, maternal age at delivery, maternal educational level, SES category, and season of LMP. A directed acyclic graph representing the proposed confounders is shown in eFigure 1 in the Supplement. The season of LMP was included as a marker of the timing of the gestation because PM_2.5_ in Mexico City has known variations by season. For assessing the change in HbA_1c_ between visits 1 and 2, the model was further adjusted for HbA_1c_ at visit 1. The results unadjusted for HbA_1c_ at visit 1 are presented in eFigure 2 in the Supplement.

### Statistical Analysis

The statistical analyses were conducted between March 11, 2018, and May 3, 2019. We fitted distributed lag nonlinear models to describe the association between prenatal PM_2.5_ exposure and HbA_1c_ at visits 1 and 2, and a normalized change in HbA_1c_ between visits 1 and 2. The data were also stratified by child sex because prenatal exposures have frequently been shown to have sex-specific effects.^14,15,24^

The estimation model was based on a generalized additive model with a penalized spline basis, according to the following formula^25^:


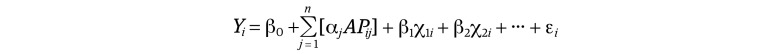
, 

where AP_ij_ is the estimated daily exposure to PM_2.5_ on day j, x_1i_, …, x_pi_ are the additional covariates for participant 1, and Y_i_ is the HbA_1c_ outcome.

The estimation models included a 392-day exposure period starting 28 days prior to LMP and ending 84 days after the estimated due date. Inclusion of this range of exposures helps increase the stability of the estimated distributed lag function through the tails of the prenatal period. Exposure was anchored at the mother’s LMP rather than the date of delivery to maintain accuracy of the developmental stage between participants; however, this means that exposures between gestational weeks 31 and 42 are prenatal for some participants and post partum for others. Results of a sensitivity analysis excluding preterm births (≤36 weeks’ gestation) is included in the eTable in the Supplement. The model was fit to allow the association of the exposure to be nonlinear at each time and to allow the associations to vary smoothly across time using the distributed lag nonlinear models package in R.^25^ Further information on distributed lag models is available in the eMethods in the Supplement. All statistical analyses were conducted in R, version 3.5.1. (R Development Core Team).

## Results

### Study Population Characteristics

Our sample included 365 children, of whom 184 were girls (50.4%) (Table). The mean (range) age of the children was approximately 4.8 (4.0-6.4) years at visit 1, and 6.7 (6.0-9.7) years at visit 2. The mean (range) time elapsed between visits was 1.9 (0.6-4.4) years. The distribution of outcome measures for HbA_1c_ at visits 1 and 2 and the time-normalized change in HbA_1c_ between visits were all approximately normal. At delivery the mothers’ mean (range) age was 27.7 (18.3-44.4) years, with a mean (range) prepregnancy BMI of 26.3 (18.5-43.5). Most children’s families were in the low category of SES (200 [54.8%]). Maternal education was evenly divided between the less than high school (145 [39.7%]), some high school or high school graduate (135 [37.0%]), and more than high school (85 [23.3%]) groups.

**Table.** Population Characteristics

Participants	All (N = 365)	Girls (n = 184)	Boys (n = 181)
Age of child at visit 1, mean (SD), y	4.78 (0.52)	4.77 (0.51)	4.79 (0.53)
Age of child at visit 2, mean (SD), y	6.71 (0.54)	6.68 (0.54)	6.73 (0.55)
Gestational age, mean (SD), mo	38.4 (1.60)	38.5 (1.55)	38.3 (1.65)
Birth weight, mean (SD), kg	3.07 (0.42)	2.99 (0.41)	3.15 (0.42)
HbA_1c_ level at visit 1, mean (SD), % of total hemoglobin^a^	5.22 (0.39)	5.24 (0.35)	5.19 (0.43)
HbA_1c_ level at visit 2, mean (SD), % of total hemoglobin^a^	5.38 (0.31)	5.39 (0.27)	5.36 (0.34)
Change in HbA_1c_ level between visits, mean (SD), % of total hemoglobin^a^	0.16 (0.47)	0.15 (0.41)	0.17 (0.52)
Mothers			
Age at delivery, mean (SD), y	27.7 (5.65)	27.3 (5.65)	28.1 (5.64)
Prepregnancy BMI, mean (SD), kg/m^2^	26.3 (4.1)	26.2 (4.3)	26.4 (3.9)
SES, No. (%)			
Low	200 (54.8)	98 (53.3)	102 (56.4)
Medium	131 (35.9)	67 (36.4)	64 (35.4)
High	34 (9.3)	19 (10.3)	15 (8.3)
Education, No. (%)			
Less than high school	145 (39.7)	67 (36.4)	78 (43.1)
High school	135 (37.0)	74 (40.2)	61 (33.7)
More than high school	85 (23.3)	43 (23.4)	42 (23.2)
Any prenatal ETS exposure, No. (%)	125 (37.7)	86 (40.6)	76 (36.9)
Season of last menstrual period			
Cold-dry (November-February), No. (%)	97 (26.6)	47 (25.5)	50 (27.6)
Warm-dry (March-April), No. (%)	76 (20.8)	40 (21.2)	36 (19.9)
Rainy (May-October), No. (%)	192 (52.6)	97 (52.7)	95 (52.5)
PM_2.5_ exposure, mean (SD), μg/m^3^	22.4 (2.7)	22.4 (2.7)	22.5 (2.6)
Preterm birth, No. (%)	37 (10.1)	19 (10.3)	18 (9.9)

Abbreviations: BMI, body mass index (calculated as weight in kilograms divided by height in meters squared); ETS, environmental tobacco smoke; HbA_1c_, hemoglobin A_1c_; PM_2.5_, particulate matter with a diameter less than 2.5 μm; SES, socioeconomic status.

^a^ SI conversion factor: To convert HbA_1c_ to proportion of total hemoglobin, multiply by 0.01.

**Figure 1.**
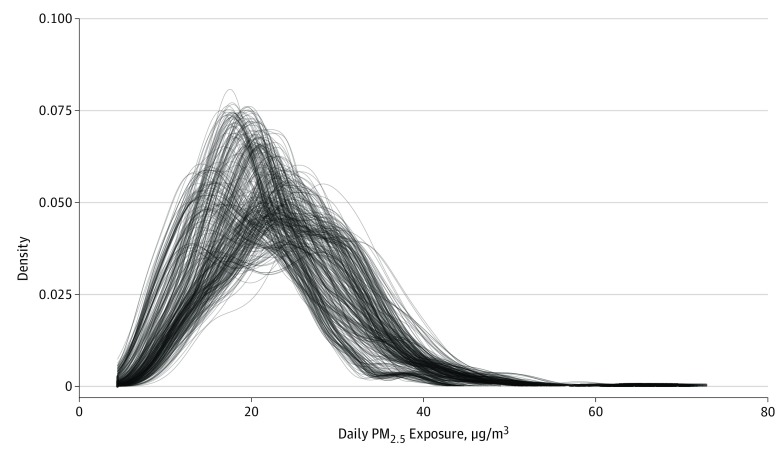
Density Plots of Estimates of Daily Exposure to Particulate Matter With Diameter Less Than 2.5 μm (PM_2.5_) for All Participants Individual daily estimates of prenatal PM_2.5_ exposure were used for each of the 365 children in the cohort, resulting in 179 144 observations. The mean (SD) PM_2.5_ exposure was 23.0 μg/m^3^ (2.7 μg/m^3^). Each continuous line in the density plot reflects 1 child’s exposures, and the density reflects the frequency of the exposure level for each child. The tails show that all children had very few days of exposure at that high level. Visual inspection of the density plots of each individual’s exposure showed no statistically significant outliers in the mean PM_2.5_ exposure.

Individual daily estimates of PM_2.5_ exposure during the study period were generated for each participant, resulting in 179 144 observations. The mean (SD) daily PM_2.5_ exposure was 22.4 μg/m^3^ (2.7 μg/m^3^). Density plots of the individual exposure measures showed no significant outliers in PM_2.5_ exposure (Figure 1).

### Exposure-Response Associations

**Figure 2.**
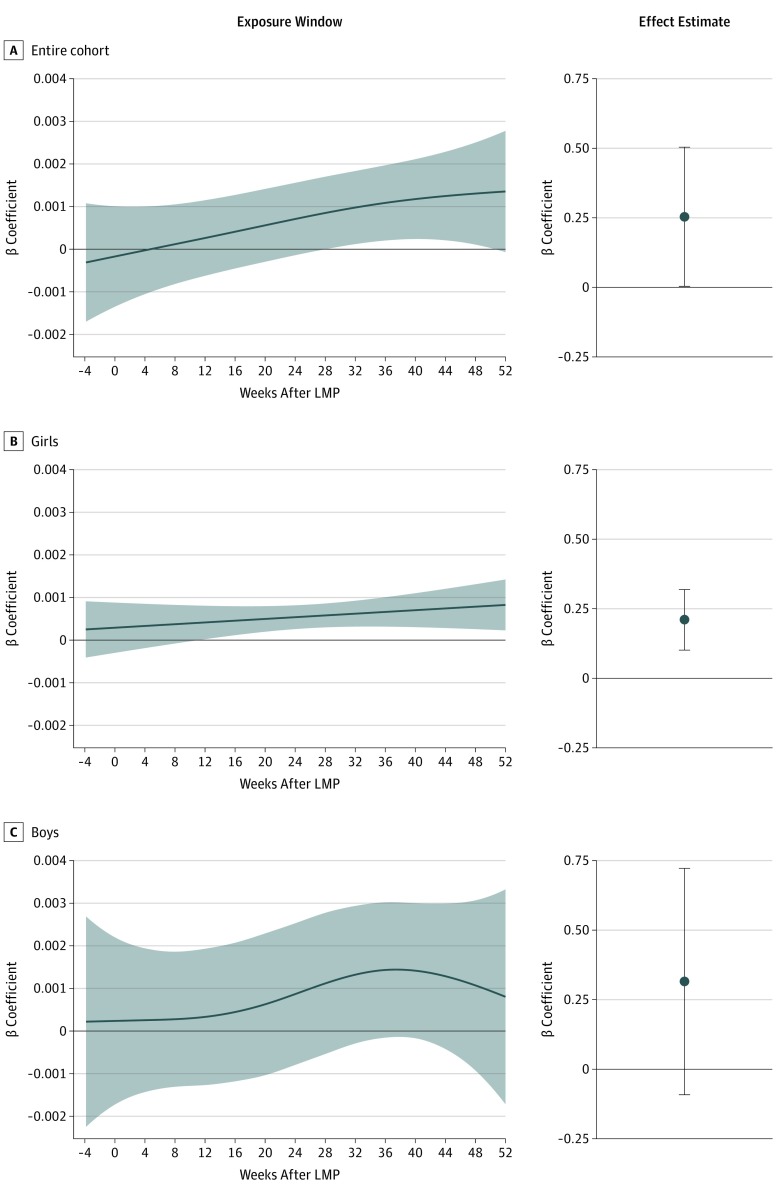
Significant Exposure Windows and Effect Estimates for Association of Exposure to Particulate Matter With Diameter Less Than 2.5 μm (PM_2.5_) With Changes in Hemoglobin A_1c_ (HbA_1c_) Levels From Visit 1 to Visit 2 Associations are based on a PM_2.5_ exposure of 23.0 μg/m^3^ compared with the cohort mean of 12 μg/m^3^. Shading indicates the 95% CIs. The x-axis of the exposure window plot depicts the week after last menstrual period (LMP) (0 is LMP), and the y-axis represents the association with HbA_1c_ level per day of PM_2.5_ exposure (percentage change per day). The graphs show the point estimate for the change in HbA_1c_ (percentage) per year of exposure to PM_2.5_. Error bars indicate 95% CIs.

The estimated association with HbA_1c_ was produced by integrating the daily effects of 23.0 μg/m^3^ of PM_2.5_ exposure throughout the study period. In the overall study population, PM_2.5_ exposure was associated with a positive rate of change in HbA_1c_ between visits 1 and 2 of 0.25% per year (95% CI, 0.004%-0.50%). Sex-stratified analyses showed a positive association between PM_2.5_ exposure and the rate of change in HbA_1c_ between visits 1 and 2 for girls (β = 0.21%; 95% CI, 0.10% to 0.32%) but not boys (β = 0.31%; 95% CI, −0.09% to 0.72%) (Figure 2).

**Figure 3.**
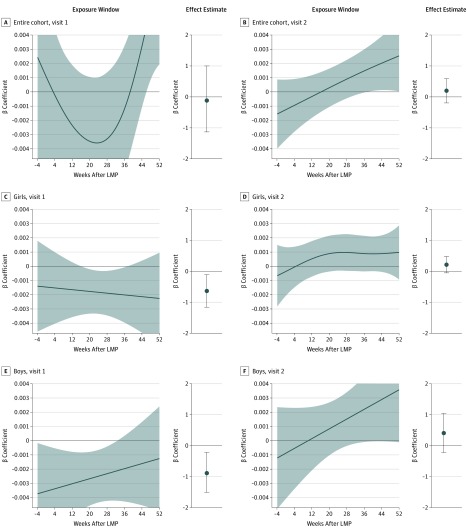
Significant Exposure Windows and Effect Estimates for Exposure to Particulate Matter With Diameter Less Than 2.5 μm (PM_2.5_) on Hemoglobin A_1c_ (HbA_1c_) Levels at Visit 1 and Visit 2 Associations are based on a PM_2.5_ exposure of 23.0 μg/m^3^ compared with 12 μg/m^3^. Shading indicates the 95% CIs. The x-axis of the exposure window plot depicts the week after the last menstrual period (LMP) (0 is LMP), and the y-axis represents the association with HbA_1c_ per day of PM_2.5_ exposure (percentage per day). The graphs show the point estimate for the association with HbA_1c_ (percentage) per year of exposure to PM_2.5_. Error bars indicate 95% CIs.

Results for HbA_1c_ at visits 1 and 2 (instead of the change over time) were dependent on child age. In the overall sample, no significant association was observed between PM_2.5_ exposure and the HbA_1c_ at visit 1 (β = −0.13%; 95% CI, −1.27% to 1.01%) or at visit 2 (β = 0.22%; 95% CI, −0.22% to 0.66%). Sex-stratified analyses showed an association between PM_2.5_ exposure and lower HbA_1c_ at visit 1 for both girls (β = −0.72%; 95% CI, −1.31% to −0.13%) and boys (β = −0.98%; 95% CI, −1.70% to −0.26%). The HbA_1c_ at visit 2 had no significant association with PM_2.5_ (girls: β = 0.24%; 95% CI, −0.05% to 0.54%; boys: β = 0.46%; 95% CI, −0.25% to 1.17%) (Figure 3). Results from a model not adjusted for baseline HbA_1c_ at visit 1 are shown in eFigure 2 and the eTable in the Supplement.

### Lag-Response Associations and Significant Exposure Windows

To derive statistically significant exposure windows, the lag-response curve was plotted for a specified exposure level, and the significant exposure window includes the period for which the 95% CI for the estimation does not include 0.

In the overall cohort, we observed a statistically significant exposure window for exposure to 23 μg/m^3^ PM_2.5_ on change in HbA_1c_ from visit 1 and visit 2 from week 16 to week 37.3 of pregnancy. The sex-stratified results showed a statistically significant exposure window for girls from week 11 of gestation through the study period but no statistically significant exposure window for boys (Figure 2).

For HbA_1c_ at visit 1, we observed statistically significant exposure windows for the overall population as well as the sex-stratified groups. For the overall cohort, there was a statistically significant exposure window from week 46 to the end of the study period. Girls had a statistically significant window from week 16 to week 37.3, and boys had a statistically significant exposure window from the beginning of the study period to week 32.7 (Figure 3).

Although the effect estimates were not statistically significant for HbA_1c_ levels at visit 2, we observed a small statistically significant exposure window for the overall cohort, from week 37.1 to the end of the study period. The sex-stratified analysis found no statistically significant exposure windows for HbA_1c_ levels at visit 2 for girls or boys (Figure 3).

## Discussion

Higher prenatal and perinatal PM_2.5_ exposure appears to be associated with glucose dysregulation in girls aged between approximately 4 to 5 years and 6 to 7 years with a critical window for exposure from the second trimester of the mother’s pregnancy to the early postnatal period. The inverse association observed at 4 to 5 years of age was not expected based on previous findings or proposed biological mechanisms.^5,7,8,9,12,26,27,28,29,30,31,32,33,34^ We hypothesize that increased prenatal and perinatal exposure to PM_2.5_ may alter glucose metabolism resulting in lower HbA_1c_ levels in early childhood (4-5 years) and higher HbA_1c_ levels in later childhood (6-7 years); however, the mechanism for these changes is unclear. For HbA_1c_ at age 4 to 5 years, we found negative associations in both boys and girls and no statistically significant association in the overall cohort. We expect that this is possible owing to the nonlinear nature of the distributed lag model, which allows the shape of associations in each dimension of the time-exposure β association curve to be driven by the data. The statistically significant windows (time vs β) show a sex difference, with a downward slope over time for girls and an upward slope for boys. In the overall sample, these associations cancelled each other out, resulting in no statistically significant association. More research is needed to replicate these findings and to more fully understand both normal and altered HbA_1c_ trajectory through childhood. In addition, prospective research on the association between prenatal and early childhood air pollution exposure and homeostatic model assessment, insulin levels, and HbA_1c_ levels could contribute to a better understanding of the mechanisms of altered glucose regulation.

### Comparison With Prior Studies

Although there is growing literature on associations between air pollution components, including PM_2.5_, and glucose dysregulation and type 2 diabetes,^5,7,8,9,12,26,27,28,29,30,31,32,33,34^ the present study provides several novel insights, including the first examination, to our knowledge, of prenatal and perinatal PM_2.5_ exposure in association with childhood HbA_1c_ changes. We characterized alterations in HbA_1c_ levels in children as young as 4 years, and our results suggested that the changes PM_2.5_ may induce may be different prior to age 4 years compared with those after age 4 years as well as by sex. Childhood type 2 diabetes is a rapidly growing area of investigation, but the current epidemiologic literature on air pollution exposure and glucose dysregulation focuses primarily on adults and may be missing important factors that occur early in life. Glucose dysregulation in childhood not only may track to later life but also may contribute to morbidity in childhood because glucose plays a key role in many developmental processes including neurodevelopment. A recent review identified 27 studies investigating air pollution exposure and diabetes or metabolic dysfunction in adults and only 6 studies on air pollution exposures and metabolic dysfunction in children published since 2012.^26^ The consensus of this literature suggests that larger exposures to air pollution contribute to glucose dysregulation and type 2 diabetes incidence. However, the evidence on prenatal exposures is sparse. Prenatal PM_2.5_ exposures have been found to be associated with increased adiposity and obesity^10,11,13^; however, we did not encounter any published literature examining prenatal air pollution exposures and glucose dysregulation or metabolic dysfunction in children.

### Biological Mechanisms

Exposure to PM_2.5_ has complex and multifactorial associations with glucose metabolism and diabetes etiology, but the proposed mechanisms, based on inflammatory and oxidative stress responses, are not completely understood.^35^ For example, when activated by PM_2.5_, bronchial epithelial cells release inflammatory mediators with downstream effects that include insulin resistance through disrupted signaling from the endothelium and liver. These mediators increase lipid peroxidation, dysregulation of visceral adipose tissue, and alterations in autonomic tone, which may further increase insulin resistance.^5,35,36^ Other proposed mechanisms work through neurologic pathways, including increased sympathetic tone and dysregulation of the hypothalamic-pituitary-adrenal axis.^35^ Prenatal PM_2.5_ exposure may cause childhood glucose dysregulation and metabolic dysfunction through similar inflammatory cascades and oxidative stress. Infants exposed to high traffic-related air pollution were shown to have exhibited reduced fetal growth and more rapid postnatal weight gain.^13^ Alterations in early life growth and childhood body weight trajectory have been associated with the risk of obesity and associated metabolic disorders including type 2 diabetes.^37^ Air pollution exposure has also been associated with gestational diabetes, or impaired glucose tolerance in pregnancy,^29,38,39,40,41^ which could be a mediator in the association between PM exposure and metabolic dysfunction in children. We were not able to evaluate this in the present cohort because information on gestational diabetes was not available. Further proposed mechanisms include metabolic dysfunction induced through alterations in the gut microbiome^42,43,44,45^ and thyroid dysfunction in pregnancy and in newborns.^46,47,48^

Prenatal exposures to air pollution have been associated with sex-dependent effects on infant lung function,^49^ childhood asthma,^50,51^ neurodevelopment,^14,52,53^ and metabolic outcomes, including childhood body composition,^15^ neonatal birth weight,^54^ and adult eating behavior as well as weight gain in animal studies.^55,56^ The mechanisms leading to sex differences in the associations of fetal exposure to PM are not well understood. Proposed mechanisms are associated with observed sex differences in fetal growth and development,^57,58^ differences in placental structural and functional development,^59^ and the effects of sex hormones.^14,57^ For example, male fetuses grow more quickly and are more likely to be larger than female fetuses, but they are also more likely to be born early and to be prone to placental dysfunction.^57^ Based on fetal sex, placentas may have different responses to some environmental chemicals.^59^ Estrogens have been shown to be anti-inflammatory through cytokine-mediated pathways^60^ and may interact with responses to proinflammatory exposures differently depending on fetal sex.

### Clinical Significance

Various biomarkers have been used to measure glucose dysregulation and metabolic dysfunction, including measures of insulin resistance such as homeostatic model assessment, β-cell function, fasting glucose, and HbA_1c_. Change in childhood HbA_1c_ level over time is a novel end point for epidemiologic investigation that requires further research and validation but offers possible benefits: it may be an early indicator of glucose dysregulation, is easily measured, and has independent clinical relevance. Glycated hemoglobin is an integrated measure of glycemic control over the previous 45 to 60 days, a diagnostic criterion for prediabetes and diabetes, and a validated measure of insulin resistance in healthy participants and in those at high risk of type 2 diabetes.^61,62^ In healthy children, the HbA_1c_ level is likely to be stable over time.^5,63^ The mean HbA_1c_ level in children in the United States has been reported to be 5.0% with small but statistically significant differences between racial or cultural groups and overweight status, but not by age.^64^ Analysis of the National Health and Nutrition Examination Survey (NHANES III)^65^ found that Mexican American children and adolescents had a mean HbA_1c_ of 5.05% compared with 4.93% in non-Hispanic white children and 5.16% in non-Hispanic black children. However, there was no statistically significant difference in HbA_1c_ levels between age groups in children aged 5 to 19 years.^64^ A longitudinal cohort of 955 healthy (nondiabetic) Dutch children found a mean annual change in HbA_1c_ levels of 0.1% per year between the ages of 8 and 12 years and was believed to be a peripubertal change that may reverse in healthy adolescents.^63^ In this context, the annual change of 0.21% to 0.25% observed in the present study may not be physiological; it may be large enough to be clinically significant.^66^ A yearly increase in HbA_1c_ of 0.25% over multiple years could mean the difference between normal (4.5%-5.6%) and prediabetes (5.7%-6.4%) or diabetes (≥6.5%). This magnitude of change has been reported in children in Sweden aged from 7 to 18 years with type 1 diabetes who had a decrease of 0.25% for 1 unit increase in physical activity.^67^ In addition, a recent observational study found that children who were obese and had a fatty liver had a higher HbA_1c_ (group mean, 5.5%) than those without fatty liver (group mean, 5.4%).^66^

Elevated HbA_1c_ may be an important indicator of insulin resistance and type 2 diabetes risk in children. Research studies have shown that high HbA_1c_ levels in children who are obese correlate with greater insulin resistance.^68^ A prospective cohort of children aged 10 to 19 years with a high risk of diabetes found that HbA_1c_ levels were associated with future diabetes risk.^69^ In addition, higher HbA_1c_ levels in both diabetic and nondiabetic populations has been associated with an increased risk of cardiovascular disease.^69,70^

These findings suggest a contributing role of air pollution in the current childhood type 2 diabetes epidemic in Mexico. The implications of these findings on the global population may be profound when considering the universal exposure to air pollution, the growing obesity epidemic, and the morbidity associated with glucose dysregulation and its sequelae.

### Strengths and Limitations

This study has several strengths. This is a prospective cohort study with the benefit of a large sample size and more than 7 years of prospective data. The use of satellite-derived data allowed us to produce individual daily exposure estimates, pinpointed to a 1 × 1-km grid that extends to before the LMP. Compared with other methods, this study provides greater spatial resolution and accuracy in Mexico City.^19^ Another strength is the application of the distributed lag nonlinear modeling, which allowed us to include the day of exposure as an independent variable and allowed the estimation of statistically significant exposure windows. Further, this nonlinear model used the data itself to drive the shape of the association, resulting in high parsimony of model fit.

This study has limitations as well. The design of this study is not causal in nature and conclusions may be made only about associations. Our exposure assessment was based on the outdoor PM_2.5_ concentrations and may not include indoor air pollution sources, although the climate of Mexico City is temperate and many homes do not have air conditioning, which often results in greater indoor-outdoor air mixing. In addition, we were not able to consider each participant’s daily movements in the city, which could contribute to further exposure misclassification. A more personalized exposure assessment, if available from the beginning of the study period, would have greater measurement accuracy; however, it also may have increased the possibility of biases from personal factors and reverse causation.^71^ Our exposure model included only PM_2.5_, but no other components of air pollution; the observed association may have been driven by another component of air pollution. We did not have information on family history of diabetes, gestational diabetes or impaired glucose tolerance in the mothers, or subsequent diabetes diagnosis in children. In addition, change in HbA_1c_ level over time in children is a novel biomarker that does not have an established clinical significance. Further research is needed to more firmly establish HbA_1c_ level ranges in healthy children and changes in the levels through childhood.

## Conclusions

To our knowledge, this is the first study of prenatal PM_2.5_ exposure and increased HbA_1c_ levels in prepubertal children. Our findings are consistent with the literature supporting a negative association of air pollution exposure with glucose metabolism in children but suggest that this association may start prior to age 4 years and may be sex specific in the period between 4 and 5 years and 6 to 7 years. The global implications of these findings may be large given the ubiquity of exposure to PM_2.5_, the morbidity associated with increased HbA_1c_ levels, and the young age of the children in the study population. Further studies on prenatal air pollution exposures and metabolic health in children and adolescents are needed to better establish the mechanisms underlying these observations and to design effective interventions to reduce risk and associated morbidity.

## Notes

Supplement 1. **eMethods.** Supplementary Methods **eFigure 1.** Directed Acyclic Graph (DAG) **eFigure 2.** Significant Exposure Windows and Effect Estimates for PM_2.5_ Exposure on Change in HbA_1c_ From Visit 1 to Visit 2, Model Unadjusted for Baseline HbA_1c_ at Visit 1 **eTable.** Findings of Sensitivity Analyses Compared to Main Findings **eReferences**
