# Supplementary material for: Association of Prenatal and Perinatal Exposures to Particulate Matter With Changes in Hemoglobin A1c Levels in Children Aged 4 to 6 Years
Source: JAMA Netw Open. 2019 Dec 18;2(12):e1917643. doi: 10.1001/jamanetworkopen.2019.17643 (PMC6991256; doi:10.1001/jamanetworkopen.2019.17643)
Supplement: Supplement 1. — eMethods. Supplementary Methods eFigure 1. Directed Acyclic Graph (DAG) eFigure 2. Significant Exposure Windows and Effect Estimates for PM2.5 Exposure on Change in HbA1c From Visit 1 to Visit 2, Model Unadjusted for Baseline HbA1c at Visit 1 eTable. Findings of Sensitivity Analyses Compared to Main Findings eReferences [file jamanetwopen-2-e1917643-s001.pdf]

# Supplementary Online Content

Moody EC, Cantoral A, Tamayao-Ortiz M, et al. Association of prenatal and perinatal exposures to particulate matter with changes in hemoglobin $A_{1c}$ levels in children aged 4 to 6 years. *JAMA Netw Open.* 2019;2(12):e1917643. doi:10.1001/jamanetworkopen.2019.17643

**eMethods.** Supplementary Methods

**eFigure 1.** Directed Acyclic Graph (DAG)

**eFigure 2.** Significant Exposure Windows and Effect Estimates for $PM_{2.5}$ Exposure on Change in $HbA_{1c}$ From Visit 1 to Visit 2, Model Unadjusted for Baseline $HbA_{1c}$ at Visit 1

**eTable.** Findings of Sensitivity Analyses Compared to Main Findings

**eReferences**

This supplementary material has been provided by the authors to give readers additional information about their work.

© 2019 Moody EC et al. *JAMA Network Open.*

## eMethods. Supplementary Methods

Distributed lag models (DLMs), initially developed as a method for time series data analysis in econometrics, have been shown to be useful in predicting the time-varying associations between prenatal environmental exposures and health outcomes[1-4]. This model for time series data is defined through a cross-basis function in which the outcome is predicted based on equally-spaced lagged values (integers) of the exposure, and describes an exposure-lag-response association. This allows for the incorporation of data from all timepoints simultaneously in the predictive model.

To generate a lag-response association describing the relationship between the exposure lag (day of $PM_{2.5}$ exposure), and the outcome (change in $HbA_{1c}$), the prediction must be made assuming constant exposure at a specified $PM_{2.5}$ level compared to another pre-specified level of $PM_{2.5}$ exposure. The specified level of exposure we chose was 23. 0 $\mu g/m^3$, which is the closest integer to the mean exposure level in the cohort (22.7 $\mu g/m^3$). Because $PM_{2.5}$ exposure is ubiquitous in the environment, comparison exposure of 0 $\mu g/m^3$ is not appropriate. We specified the comparison exposure level of 12 $\mu g/m^3$. This number represents both the Mexican Air Quality Standard, and the primary standard for $PM_{2.5}$ that was set by the US EPA in 1990 as part of the National Ambient Air Quality Standards (NAAQS). The Mexican National Standard is a regulatory standard, defined by the Secretary of Health and implemented in 1994. It is an annual standard defined as the 75$^{th}$ percentile of each 24-hour concentration per trimester, averaged over one year or at least three trimesters[5]. The US NAAQS standard is an annual mean intended to protect the public health[6].

© 2019 Moody EC et al. *JAMA Network Open.*

## eFigure 1. Directed Acyclic Graph (DAG)

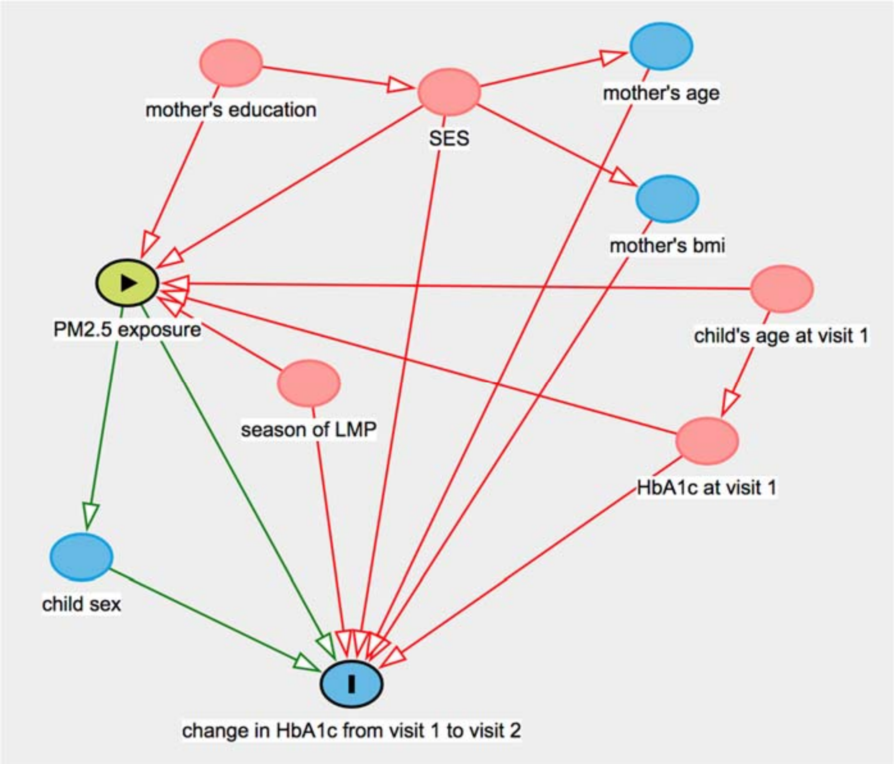


Directed acyclic graphs are a way of representing causal relationships, and can be used to identify appropriate confounders to avoid confounding bias. Green arrows indicate causal paths, red arrows indicate biasing paths. Blue variables are associated with the outcome, red variables are associated with the exposure and the outcome. Created using DAGitty[7]

© 2019 Moody EC et al. *JAMA Network Open.*

**eFigure 2. Significant Exposure Windows and Effect Estimates for $PM_{2.5}$ Exposure on Change in $HbA_{1c}$ From Visit 1 to Visit 2, Model Unadjusted for Baseline $HbA_{1c}$ at Visit 1**

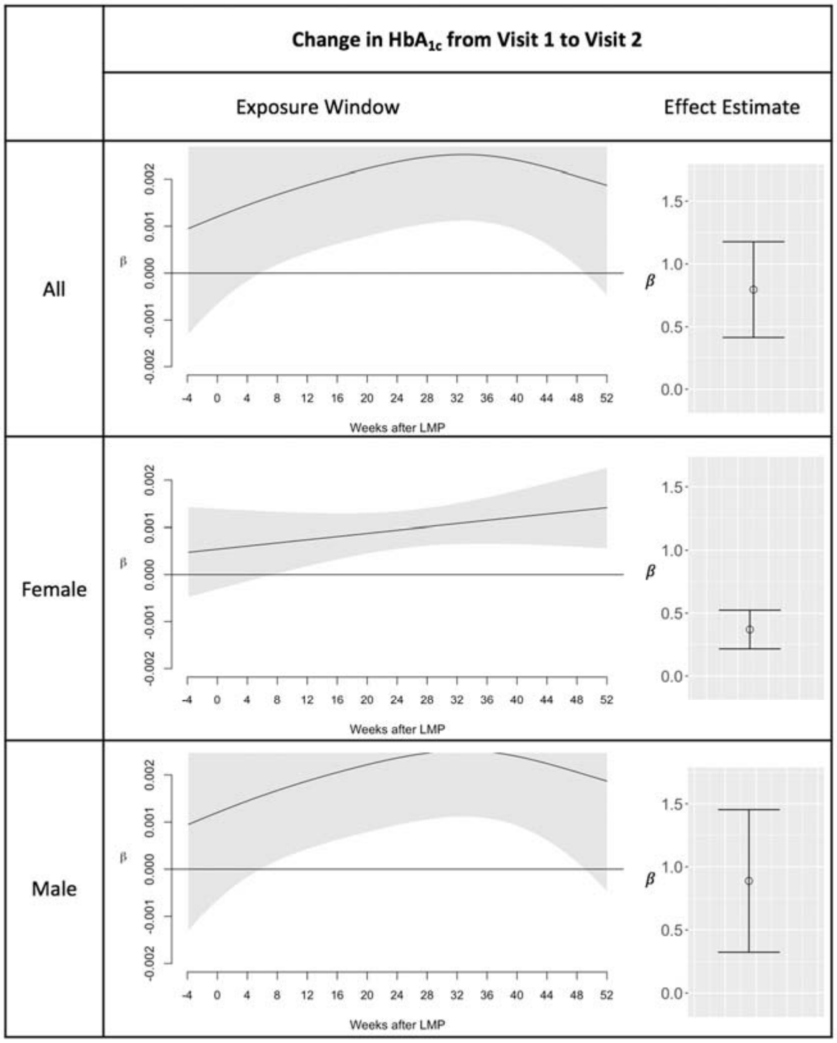


Caption: Predictions are based upon an exposure of 23 μg/m$^3$, compared to 12 μg/m$^3$. The x-axis is the week after the day of mother's last menstrual period (LMP) (0 is LMP), and the y-axis is effect on $HbA_{1c}$ per day of $PM_{2.5}$ exposure (% change per day). In the overall study population, $PM_{2.5}$ exposure was associated with a positive rate of change of $HbA_{1c}$ between visit 1 and 2 of 0.80% per year (95% CI 0.42, 1.18). Sex-stratified analyses showed positive association between $PM_{2.5}$ exposure and the rate of change of $HbA_{1c}$ between visit 1 and 2 for female (β = 0.43, 95% CI: 0.17, 0.68) and male (β = 0.89, 95% CI: 0.32, 1.45) children. In the overall cohort we observed a significant exposure window from week 5.9 to week 49 post conception. The sex-stratified results showed a significant exposure window for female children from week 11.7 of gestation through the study period and from week 6 to week 45.6 for male children.

© 2019 Moody EC et al. *JAMA Network Open.*

# eTable. Findings of Sensitivity Analyses Compared to Main Findings

| | Outcome | Group (n) | | ß (95% CI) | Significant exposure window (weeks after LMP) |
|---|---|---|---|---|---|
| Main Finding | Change in HbA1c from visit 1 to visit 2 | Overall (n=458) | | **0.25% (0.004, 0.50)** | 28.0 - 50.6 |
| | | Female (n=230) | | **0.21% (0.10, 0.32)** | 11.0 - 52.0 |
| | | Male (n=228) | | 0.31% (-0.09, 0.72) | none |
| Secondary findings | HbA1c at visit 1 | Overall (n=458) | | -0.13% (-1.27, 1.01) | 46.0 - 52.0 |
| | | Female (n=230) | | **-0.72% (-1.31, -0.13)** | 16.0 - 37.3 |
| | | Male (n=228) | | **-0.98% (-1.70, -0.26)** | -4.0 - 32.7 |
| | HbA1c at visit 2 | Overall (n=458) | | 0.22% (-0.22, 0.66) | 37.1 - 52.0 |
| | | Female (n=230) | | 0.24% (-0.05, 0.54) | none |
| | | Male (n=228) | | 0.46% (-0.25, 1.17) | none |
| Sensitivity analysis: Unadjusted for HbA1c at visit 1 | Change in HbA1c from visit 1 to visit 2 | Overall (n=458) | | **0.80% (0.42, 1.18)** | 5.7 - 49.0 |
| | | Female (n=230) | | **0.37% (0.22, 0.52)** | 7.3 - 52 |
| | | Male (n=228) | | **0.89% (0.32, 1.45)** | 5.9 - 45.6 |
| Sensitivity analysis: Exclude preterm births (≤36 weeks) | Change in HbA1c from visit 1 to visit 2 | Overall (n=414) | | **0.29% (0.03, 0.55)** | 22.6 - 52.0 |
| | | Female (n=208) | | **0.24% (0.13, 0.36)** | 10.6 - 52.0 |
| | | Male (n=206) | | **0.50% (0.06, 0.95)** | 21.4 - 52.0 |
| | HbA1c at visit 1 | Overall (n=414) | | -0.34% (-1.54, 0.87) | 47.6 - 52.0 |
| | | Female (n=208) | | **-0.80% (-1.46, -0.14)** | 16.9 - 38.7 |
| | | Male (n=206) | | **-0.90% (-1.64, -0.17)** | -3.1 - 30.4 |
| | HbA1c at visit 2 | Overall (n=414) | | 0.24% (-0.24, 0.71) | 33.6 - 52.0 |
| | | Female (n=208) | | 0.21% (-0.17, 0.60) | none |
| | | Male (n=206) | | 0.61% (-0.17, 1.39) | 29.4 - 52.0 |
| Sensitivity analysis: Stratified by ETS exposure | Change in HbA1c from visit 1 to visit 2 | Exposed to ETS | Overall (n=162) | -0.02% (-0.19, 0.14) | 11.1 - 16.1, 31.4 - 35.4 |
| | | | Female (n=86) | * | * |
| | | | Male (n=76) | * | * |
| | Change in HbA1c from visit 1 to visit 2 | No ETS exposure | Overall (n=256) | **0.21% (0.05, 0.37)** | 13.0 - 35.9 |
| | | | Female (n=126) | 0.19% (-0.09, 0.47) | none |

© 2019 Moody EC et al. *JAMA Network Open.*

| | | | | | |
|---|---|---|---|---|---|
| | | | Male (n=130) | 0.29% (-0.03, 0.61) | none |
| | HbA1c at visit 1 | Exposed to ETS | Overall (n=162) | **-0.65% (-1.25, -0.05)** | 20.9 - 34.7 |
| | | | Female (n=86) | * | * |
| | | | Male (n=76) | * | * |
| | HbA1c at visit 1 | No ETS exposure | Overall (n=256) | -0.08% (-1.40, 1.24) | 45.9 - 52.0 |
| | | | Female (n=126) | **-0.40% (-0.73, -0.08)** | 13.4 - 34.1 |
| | | | Male (n=130) | -1.15% (-2.99, 0.69) | none |
| | HbA1c at visit 2 | Exposed to ETS | Overall (n=162) | -0.13% (-0.44, 0.17) | none |
| | | | Female (n=86) | * | * |
| | | | Male (n=76) | * | * |
| | HbA1c at visit 2 | No ETS exposure | Overall (n=256) | 0.20% (-0.24, 0.63) | none |
| | | | Female (n=126) | 0.08% (-0.50, 0.65) | none |
| | | | Male (n=130) | 0.45% (-0.20, 1.09) | none |

* insufficient sample size to complete the analysis

statistically significant results highlighted in bold

We performed three sensitivity analyses. The first sensitivity analysis did not adjust the model for the outcome of change in HbA1c from visit 1 to visit 2 for the HbA1c at visit 1. The second analysis excluded preterm births (born at ≤36 weeks gestation). The third analysis stratified the participants by presence of prenatal environmental tobacco smoke (ETS) exposure. The sample sizes were small after a second stratification for the third analysis and the model was not able to run in all cases.

© 2019 Moody EC et al. *JAMA Network Open.*

## eReferences


1. Rosa MJ, Just AC, Guerra MS, et al. Identifying sensitive windows for prenatal particulate air pollution exposure and mitochondrial DNA content in cord blood. *Environ Int.* 2017;98:198-203.
2. Chiu YH, Hsu HH, Coull BA, et al. Prenatal particulate air pollution and neurodevelopment in urban children: Examining sensitive windows and sex-specific associations. *Environ Int.* 2016;87:56-65.
3. Chiu YH, Hsu HH, Wilson A, et al. Prenatal particulate air pollution exposure and body composition in urban preschool children: Examining sensitive windows and sex-specific associations. *Environ Res.* 2017;158:798-805.
4. Martens DS, Cox B, Janssen BG, et al. Prenatal Air Pollution and Newborns' Predisposition to Accelerated Biological Aging. *JAMA pediatrics.* 2017;171(12):1160-1167.
5. TransportPolicy.net. Mexico: Air Quality Standards. https://www.transportpolicy.net/standard/mexico-air-quality-standards/ Published 2018. Accessed November 5, 2018.
6. Agency USEP. NAAQS Table. https://www.epa.gov/criteria-air-pollutants/naaqs-table. Updated December 20, 2016. Accessed January 14, 2019.
7. Textor J, van der Zander B, Gilthorpe MS, Liskiewicz M, Ellison GT. Robust causal inference using directed acyclic graphs: the R package 'dagitty'. *Int J Epidemiol.* 2016;45(6):1887-1894.


© 2019 Moody EC et al. *JAMA Network Open.*
